# 3,4-Dimethyl­anilinium chloride monohydrate

**DOI:** 10.1107/S1600536809006072

**Published:** 2009-02-28

**Authors:** Sofiane Bouacida, Ratiba Belhouas, Habiba Kechout, Hocine Merazig, Patricia Bénard-Rocherullé

**Affiliations:** a Département de Chimie, Faculté des Sciences et Sciences de l’Ingénieur, Université A. Mira de Béjaia, Route Targua Ouzmour, 06000 Béjaia, Algeria; b Laboratoire de Chimie Moléculaire du Contrôle de l’Environnement et des Mesures Physico-Chimiques, Faculté des Sciences Exactes, Département de Chimie, Université Mentouri, Constantine 25000, Algeria; c Faculté de Chimie, USTHB, BP32, El-Alia, Bab-Ezzouar, Alger, Algeria; d Sciences Chimiques de Rennes (UMR CNRS 6226), Université de Rennes 1, Avenue du Général Leclerc, 35042 Rennes Cedex, France

## Abstract

The crystal structure of the title compound, C_8_H_12_N^+^·Cl^−^·H_2_O, consists of hydro­phobic layers of dimethyl­anilinium cations parallel to the *bc* plane alternated by hydro­philic layers of chloride anions and water mol­ecules. The layers are linked by N—H⋯O and N—H⋯Cl hydrogen bonds involving the ammonium groups of the cations. The cohesion of the ionic structure is further stabilized by O—H⋯Cl hydrogen-bonding inter­actions.

## Related literature

For crystal structures containing the dimethyl­anilinium cation, see: Bouacida (2008 ▶); Singh *et al.* (2002 ▶); Singh *et al.* (1995*a* ▶,*b* ▶); Linden *et al.* (1995 ▶); Fábry *et al.* (2001 ▶, 2002 ▶). For the crystal structures of related protonated amines, see: Bouacida *et al.* (2005*a* ▶,*b* ▶,*c* ▶, 2006 ▶, 2007 ▶); Benslimane *et al.* (2007 ▶); Rademeyer (2004*a* ▶,*b* ▶).
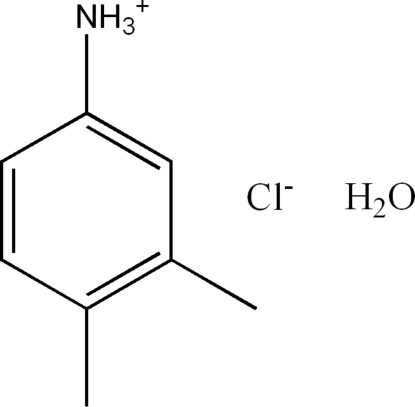


## Experimental

### 

#### Crystal data

C_8_H_12_N^+^·Cl^−^·H_2_O
*M* *_r_* = 175.65
Orthorhombic, 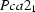

*a* = 18.230 (18) Å
*b* = 6.7854 (14) Å
*c* = 7.916 (2) Å
*V* = 979.2 (10) Å^3^
*Z* = 4
Mo *K*α radiation
μ = 0.34 mm^−1^
*T* = 295 K
0.1 × 0.04 × 0.02 mm

#### Data collection

Enraf–Nonius KappaCCD diffractometer
Absorption correction: none
10115 measured reflections
2181 independent reflections
1403 reflections with *I* > 2σ(*I*)
*R*_int_ = 0.078

#### Refinement

*R*[*F* ^2^ > 2σ(*F* ^2^)] = 0.059
*wR*(*F* ^2^) = 0.109
*S* = 1.15
2181 reflections
109 parameters
1 restraint
H atoms treated by a mixture of independent and constrained refinement
Δρ_max_ = 0.20 e Å^−3^
Δρ_min_ = −0.22 e Å^−3^
Absolute structure: Flack (1983 ▶), 976 Friedel pairs
Flack parameter: 0.01 (11)

### 

Data collection: *COLLECT* (Nonius, 1998 ▶); cell refinement: *SCALEPACK* (Otwinowski & Minor, 1997 ▶); data reduction: *DENZO* (Otwinowski & Minor, 1997 ▶); and *SCALEPACK* program(s) used to solve structure: *SIR2002* (Burla *et al.*, 2003 ▶); program(s) used to refine structure: *SHELXL97* (Sheldrick, 2008 ▶); molecular graphics: *ORTEP-3* (Farrugia, 1997 ▶) and *DIAMOND* (Brandenburg *et al.*, 2001 ▶); software used to prepare material for publication: *WinGX* (Farrugia, 1999 ▶).

## Supplementary Material

Crystal structure: contains datablocks global, I. DOI: 10.1107/S1600536809006072/rz2296sup1.cif

Structure factors: contains datablocks I. DOI: 10.1107/S1600536809006072/rz2296Isup2.hkl

Additional supplementary materials:  crystallographic information; 3D view; checkCIF report

## Figures and Tables

**Table 1:** Hydrogen-bond geometry (Å, °)

*D*—H⋯*A*	*D*—H	H⋯*A*	*D*⋯*A*	*D*—H⋯*A*
N1—H1*A*⋯O1*W*	0.89	1.87	2.754 (5)	174
N1—H1*B*⋯Cl1^i^	0.89	2.30	3.177 (4)	167
N1—H1*C*⋯Cl1^ii^	0.89	2.31	3.181 (4)	167
O1*W*—H1*W*⋯Cl1	0.80 (6)	2.43 (6)	3.217 (5)	174 (7)
O1*W*—H2*W*⋯Cl1^iii^	0.81 (5)	2.36 (5)	3.174 (5)	176 (2)

Symmetry codes: (i) 
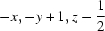
; (ii) 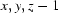; (iii) 
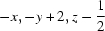
.

**Fig. 1.**
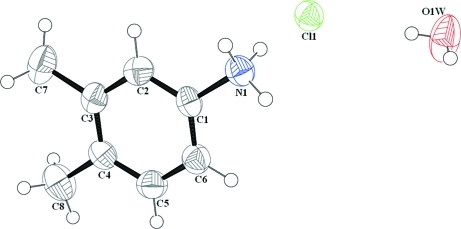
The structure of the title compound with the atomic labelling scheme. Displacement ellipsoids are drawn at the 50% probability level.

**Fig. 2.**
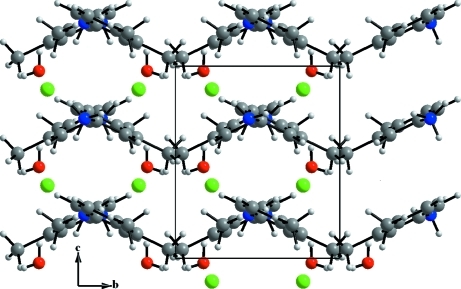
A diagram of the layered crystal packing in the title comound, viewed down the a axis.

**Fig. 3.**
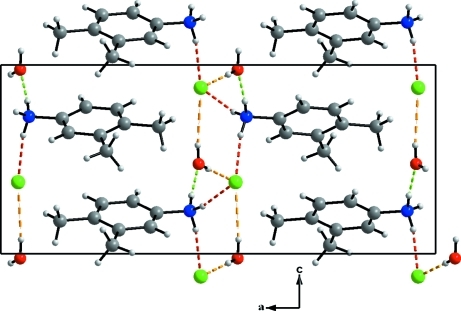
Crystal packing of the title compound viewed down the b axis. H bonds are shown as dashed lines.

## supplementary crystallographic information

### Comment

The title compound, was prepared as part of our ongoing studies of hydrogen-bonding interactions in the crystal structure of protonated amines (Bouacida *et al.*, 2005*a*,b,c; Bouacida *et al.*, 2006; Benslimane *et al.*, 2007; Bouacida *et al.*, 2007). Structures containing the dimethylanilinium cation have been already reported with tin chloride (Bouacida, 2008), sulfate (Singh *et al.*, 2002), nitrate and perchlorate (Singh *et al.*, 1995*a*,b), chloride (Linden *et al.*, 1995), and dihydrogenphosphate (Fabry *et al.*, 2001; Fábry *et al.*, 2002).

The molecular structure of the title compound is illustrated in Fig. 1. A l l bond distances and angles are within the ranges of accepted values. The amino N atom is protonated as in other aminoacids (Bouacida *et al.*, 2006; Rademeyer 2004*a*,b). A diagram of the layered crystal packing of title compound is shown in Fig. 2, in which the cations are arranged to form zigzag layers parallel the *ab* plane, with the chloride ions and water molecules located between these layers. The structure may be also described as formed by hydrophobic layers parallel to the *bc* plane of dimethylanilinium cations alternated by hydrophilic layers of chloride anions and water molecules. In this structure, three types of classical hydrogen bonds are observed, *viz*. cation–anion, cation–water and water–anion (Fig. 3, Table 1). All three ammonium H atoms are involved in hydrogen bonds. These interactions link the molecules within the layers and also link the layers together, forming a three-dimensional network and reinforcing the cohesion of the ionic structure.

### Experimental

An aqueous solution of SnCl_2_.2H_2_O (1 mmol) and 3,4-dimethylaniline (2 mmol) in hydrochloric acid was slowly evaporated to dryness for two weeks. White single crystals of the title compound were carefully isolated under polarizing microscope for X-ray diffraction analysis

### Refinement

The water H atoms were located in a difference Fourier map and refined isotropically, with *U*_iso_(H) =1.25(O). All other H atoms were localized in difference Fourier maps but introduced in calculated positions and treated as riding on their parent atoms, with C—H = 0.93–0.96 Å, N—H = 0.89Å and *U*_iso_(H) = 1.2 *U*_eq_(C, N) or 1.5 *U*_eq_(C) for methyl H atoms.

### Crystal data

**Table:**

C_8_H_12_N^+^·Cl^−^·H_2_O	*F*(000) = 376
*M**_r_* = 175.65	*D*_x_ = 1.191 Mg m^−^^3^
Orthorhombic, *P**c**a*2_1_	Mo *K*α radiation, λ = 0.71073 Å
Hall symbol: P 2c -2ac	Cell parameters from 9401 reflections
*a* = 18.230 (18) Å	θ = 3.7–27.5°
*b* = 6.7854 (14) Å	µ = 0.34 mm^−^^1^
*c* = 7.916 (2) Å	*T* = 295 K
*V* = 979.2 (10) Å^3^	Stalk, white
*Z* = 4	0.1 × 0.04 × 0.02 mm

### Data collection

**Table:**

Enraf–Nonius KappaCCD diffractometer	*R*_int_ = 0.078
CCD rotation images, thick slices scans	θ_max_ = 27.5°, θ_min_ = 3.7°
10115 measured reflections	*h* = −23→23
2181 independent reflections	*k* = −8→8
1403 reflections with *I* > 2σ(*I*)	*l* = −10→9

### Refinement

**Table:**

Refinement on *F*^2^	H atoms treated by a mixture of independent and constrained refinement
Least-squares matrix: full	*w* = 1/[σ^2^(*F*_o_^2^) + (0.0307*P*)^2^ + 0.3106*P*] where *P* = (*F*_o_^2^ + 2*F*_c_^2^)/3
*R*[*F*^2^ > 2σ(*F*^2^)] = 0.059	(Δ/σ)_max_ < 0.001
*wR*(*F*^2^) = 0.109	Δρ_max_ = 0.20 e Å^−^^3^
*S* = 1.15	Δρ_min_ = −0.21 e Å^−^^3^
2181 reflections	Absolute structure: Flack (1983), 976 Friedel pairs
109 parameters	Flack parameter: 0.01 (11)
1 restraint	

### Special details

**Table:**

Geometry. Bond distances, angles *etc*. have been calculated using the rounded fractional coordinates. All su's are estimated from the variances of the (full) variance-covariance matrix. The cell e.s.d.'s are taken into account in the estimation of distances, angles and torsion angles

### Fractional atomic coordinates and isotropic or equivalent isotropic displacement parameters (Å^2^)

**Table:**

	*x*	*y*	*z*	*U*_iso_*/*U*_eq_	
N1	0.06494 (14)	0.5513 (4)	0.2268 (4)	0.0433 (10)	
C1	0.14146 (17)	0.4809 (5)	0.2159 (4)	0.0395 (11)	
C2	0.1556 (2)	0.3036 (6)	0.1426 (5)	0.0447 (12)	
C3	0.22856 (19)	0.2362 (5)	0.1269 (4)	0.0430 (13)	
C4	0.28449 (18)	0.3555 (5)	0.1893 (5)	0.0444 (11)	
C5	0.2677 (2)	0.5332 (6)	0.2641 (5)	0.0517 (14)	
C6	0.1959 (2)	0.5966 (5)	0.2776 (5)	0.0467 (12)	
C7	0.2432 (3)	0.0413 (6)	0.0454 (6)	0.0670 (19)	
C8	0.3636 (2)	0.2894 (7)	0.1744 (7)	0.0693 (16)	
O1W	0.0447 (3)	0.8297 (5)	0.4757 (4)	0.0818 (13)	
Cl1	0.04002 (5)	0.77733 (12)	0.87943 (11)	0.0507 (3)	
H1A	0.06154	0.64300	0.30678	0.0650*	
H1B	0.03559	0.45076	0.25217	0.0650*	
H1C	0.05158	0.60253	0.12795	0.0650*	
H2	0.11712	0.22655	0.10264	0.0534*	
H5	0.30525	0.61175	0.30623	0.0620*	
H6	0.18505	0.71675	0.32834	0.0559*	
H7A	0.27619	0.05896	−0.04794	0.1007*	
H7B	0.19792	−0.01381	0.00532	0.1007*	
H7C	0.26488	−0.04643	0.12637	0.1007*	
H8A	0.39537	0.39319	0.21227	0.1038*	
H8B	0.37436	0.25867	0.05870	0.1038*	
H8C	0.37112	0.17444	0.24296	0.1038*	
H1W	0.046 (3)	0.824 (9)	0.576 (7)	0.1038*	
H2W	0.022 (3)	0.927 (8)	0.447 (7)	0.1038*	

### Atomic displacement parameters (Å^2^)

**Table:**

	*U*^11^	*U*^22^	*U*^33^	*U*^12^	*U*^13^	*U*^23^
N1	0.0455 (16)	0.0461 (17)	0.0383 (18)	0.0025 (13)	−0.0007 (14)	0.0048 (14)
C1	0.0384 (18)	0.044 (2)	0.0361 (19)	0.0062 (16)	0.0041 (17)	0.0089 (18)
C2	0.051 (2)	0.043 (2)	0.040 (2)	−0.0076 (17)	0.0008 (19)	0.0070 (18)
C3	0.060 (3)	0.039 (2)	0.0300 (17)	0.0022 (17)	0.004 (2)	0.0089 (17)
C4	0.047 (2)	0.051 (2)	0.0353 (19)	0.0013 (17)	0.0013 (19)	0.010 (2)
C5	0.052 (2)	0.051 (2)	0.052 (3)	−0.0056 (18)	−0.006 (2)	0.002 (2)
C6	0.049 (2)	0.045 (2)	0.046 (2)	0.0024 (18)	−0.0018 (19)	0.0034 (18)
C7	0.097 (4)	0.048 (3)	0.056 (3)	0.012 (2)	0.008 (3)	0.000 (2)
C8	0.052 (2)	0.082 (3)	0.074 (3)	0.009 (2)	0.007 (3)	0.011 (3)
O1W	0.127 (3)	0.066 (2)	0.0523 (18)	0.037 (2)	−0.004 (2)	−0.0054 (17)
Cl1	0.0555 (4)	0.0492 (5)	0.0475 (4)	0.0042 (4)	−0.0028 (6)	0.0044 (6)

### Geometric parameters (Å, °)

**Table:**

O1W—H2W	0.81 (5)	C4—C5	1.378 (6)
O1W—H1W	0.80 (6)	C5—C6	1.382 (5)
N1—C1	1.477 (4)	C2—H2	0.9300
N1—H1A	0.8900	C5—H5	0.9300
N1—H1C	0.8900	C6—H6	0.9300
N1—H1B	0.8900	C7—H7B	0.9600
C1—C2	1.360 (5)	C7—H7C	0.9600
C1—C6	1.356 (5)	C7—H7A	0.9600
C2—C3	1.412 (5)	C8—H8C	0.9600
C3—C7	1.496 (6)	C8—H8A	0.9600
C3—C4	1.392 (5)	C8—H8B	0.9600
C4—C8	1.515 (5)		
			
Cl1···O1W	3.217 (5)	C8···H7A	2.8400
Cl1···N1^i^	3.181 (4)	H1A···H2W	2.3400
Cl1···N1^ii^	3.177 (4)	H1A···O1W	1.8700
Cl1···O1W^iii^	3.174 (5)	H1A···H6	2.3100
Cl1···H1W	2.43 (6)	H1A···H1W	2.4800
Cl1···H1C^i^	2.3100	H1B···Cl1^vi^	2.3000
Cl1···H1B^ii^	2.3000	H1B···H2	2.4300
Cl1···H5^iv^	3.0900	H1C···Cl1^vii^	2.3100
Cl1···H2W^iii^	2.36 (5)	H1W···H1A	2.4800
O1W···Cl1^v^	3.174 (5)	H1W···Cl1	2.43 (6)
O1W···N1	2.754 (5)	H2···H1B	2.4300
O1W···Cl1	3.217 (5)	H2···H7B	2.3300
O1W···H1A	1.8700	H2W···Cl1^v^	2.36 (5)
O1W···H6	2.9100	H2W···H1A	2.3400
N1···Cl1^vi^	3.177 (4)	H5···H8A	2.3300
N1···Cl1^vii^	3.181 (4)	H5···Cl1^viii^	3.0900
N1···O1W	2.754 (5)	H6···H1A	2.3100
C3···C5^viii^	3.509 (6)	H6···C7^xi^	3.0800
C3···C4^viii^	3.565 (6)	H6···O1W	2.9100
C4···C3^iv^	3.565 (6)	H7A···H8B	2.4000
C4···C7^iv^	3.570 (7)	H7A···C8	2.8400
C5···C3^iv^	3.509 (6)	H7A···C3^viii^	2.8400
C7···C4^viii^	3.570 (7)	H7A···C4^viii^	3.1000
C3···H7A^iv^	2.8400	H7B···H2	2.3300
C4···H7A^iv^	3.1000	H7C···C8	2.9300
C5···H7C^ix^	3.0500	H7C···C5^xii^	3.0500
C6···H7C^ix^	2.9800	H7C···C6^xii^	2.9800
C7···H8B	2.8100	H8A···H5	2.3300
C7···H8C	2.9500	H8B···C7	2.8100
C7···H6^x^	3.0800	H8B···H7A	2.4000
C8···H7C	2.9300	H8C···C7	2.9500
			
H1W—O1W—H2W	110 (6)	C3—C2—H2	120.00
H1A—N1—H1B	109.00	C1—C2—H2	120.00
H1A—N1—H1C	109.00	C4—C5—H5	120.00
C1—N1—H1B	109.00	C6—C5—H5	119.00
C1—N1—H1C	109.00	C5—C6—H6	121.00
H1B—N1—H1C	109.00	C1—C6—H6	120.00
C1—N1—H1A	109.00	C3—C7—H7A	109.00
N1—C1—C2	119.3 (3)	C3—C7—H7B	109.00
C2—C1—C6	121.8 (3)	H7A—C7—H7B	109.00
N1—C1—C6	118.9 (3)	H7A—C7—H7C	109.00
C1—C2—C3	120.2 (3)	C3—C7—H7C	110.00
C2—C3—C4	118.1 (3)	H7B—C7—H7C	109.00
C2—C3—C7	119.5 (4)	C4—C8—H8B	109.00
C4—C3—C7	122.4 (3)	C4—C8—H8C	109.00
C3—C4—C8	119.8 (3)	C4—C8—H8A	109.00
C5—C4—C8	120.3 (3)	H8A—C8—H8C	109.00
C3—C4—C5	119.9 (3)	H8B—C8—H8C	109.00
C4—C5—C6	121.1 (3)	H8A—C8—H8B	110.00
C1—C6—C5	119.0 (3)		
			
N1—C1—C2—C3	178.3 (3)	C2—C3—C4—C8	−179.7 (4)
C6—C1—C2—C3	−0.9 (6)	C7—C3—C4—C5	−179.6 (4)
N1—C1—C6—C5	−178.5 (3)	C7—C3—C4—C8	0.5 (6)
C2—C1—C6—C5	0.6 (6)	C3—C4—C5—C6	−0.4 (6)
C1—C2—C3—C4	0.4 (5)	C8—C4—C5—C6	179.5 (4)
C1—C2—C3—C7	−179.8 (4)	C4—C5—C6—C1	0.0 (6)
C2—C3—C4—C5	0.2 (5)		

Symmetry codes: (i) *x*, *y*, *z*+1; (ii) −*x*, −*y*+1, *z*+1/2; (iii) −*x*, −*y*+2, *z*+1/2; (iv) −*x*+1/2, *y*, *z*+1/2; (v) −*x*, −*y*+2, *z*−1/2; (vi) −*x*, −*y*+1, *z*−1/2; (vii) *x*, *y*, *z*−1; (viii) −*x*+1/2, *y*, *z*−1/2; (ix) *x*, *y*+1, *z*; (x) −*x*+1/2, *y*−1, *z*−1/2; (xi) −*x*+1/2, *y*+1, *z*+1/2; (xii) *x*, *y*−1, *z*.

### Hydrogen-bond geometry (Å, °)

**Table:**

*D*—H···*A*	*D*—H	H···*A*	*D*···*A*	*D*—H···*A*
N1—H1A···O1W	0.8900	1.8700	2.754 (5)	174.00
N1—H1B···Cl1^vi^	0.8900	2.3000	3.177 (4)	167.00
N1—H1C···Cl1^vii^	0.8900	2.3100	3.181 (4)	167.00
O1W—H1W···Cl1	0.80 (6)	2.43 (6)	3.217 (5)	174 (7)
O1W—H2W···Cl1^v^	0.81 (5)	2.36 (5)	3.174 (5)	176 (2)

Symmetry codes: (vi) −*x*, −*y*+1, *z*−1/2; (vii) *x*, *y*, *z*−1; (v) −*x*, −*y*+2, *z*−1/2.
